# Response Model for Urban Area Source Pollution and Water Environmental Quality in a River Network Region

**DOI:** 10.3390/ijerph191710546

**Published:** 2022-08-24

**Authors:** Qiuying Lai, Jie Ma, Fei He, Geng Wei

**Affiliations:** 1 Nanjing Institute of Environmental Sciences, Ministry of Ecology and Environment, Nanjing 210042, China; 2 College of Harbour, Coastal and Offshore Engineering, Hohai University, Nanjing 210098, China

**Keywords:** urban area source pollution, water environmental quality, model coupling, plain river network, Yangtze River Delta of China

## Abstract

With the development of cities, urban area source pollution has become more severe and a significant source of water pollution. To study the relationship between urban area source pollution and water environmental quality in a river network, this study uses a city in the Yangtze River Delta, China, as an example. The Storm Water Management Model (SWMM) model and the MIKE11 model were combined into a unified modeling framework and used to simulate dynamic changes in the water quality of a river network under light rain, moderate rain, and heavy rain. In the study period, the annual urban area source input loads of potassium permanganate (COD_Mn_), total phosphorus (TP), and ammonia nitrogen were 29.8, 0.9, and 4.8 t, respectively. The influence of light rain on the water quality of the river network was lagging and temporary, and rainfall area pollution was the primary contributor. Under the scenario of moderate rain, overflow from a pipeline network compounded rainfall runoff, resulting in a longer duration of impact on the water quality in the river. Additionally, the water quality in the river course was worse under moderate rain than under light or heavy rain. Under the scenario of heavy rain, rain mainly served a dilutive function. This research can provide support for urban area source pollution control and management.

## 1. Introduction

Water pollution is a worldwide concern [1], and area source pollution has become the world’s leading source of water pollution [2]. Area source water pollution occurs in a multifaceted manner and is involved in several dynamic processes, including rainfall runoff, soil erosion, pollutant accumulation, and leaching of soil solutes. Area source pollution is random, complex, uncertain, and characterized by hysteresis [3,4,5]. With rapid urbanization, the proportion of impervious surfaces in downtown areas has increased. When excessive rainfall occurs in metropolitan areas, it can exceed the capacity of drainage systems, resulting in poor management of the urban water environment [5,6]. Urban area source pollution resulting from storm water has been identified as a major cause of water quality degradation [7]. Previous studies have shown that urban area source pollution comprises up to 50% of the water pollution in the central urban areas in Beijing and Shanghai [8]. In recent years, urban area source pollution studies have mainly focused on pollutant buildup and washoff processes, the first flush effect, pollutant concentrations, best management practices, and the total pollutant mass in cities during rainfall events [9]. Because many biological processes are fundamentally dependent on the hydrological cycle, ecosystem health is closely tied to water quality. However, the mechanisms are not fully understood. To protect environmental water quality in urban areas, a comprehensive and multi-method management strategy will be required.

Managing water environmental quality is a complicated challenge, and the conventional decision-making process is inevitably subjective and unilateral. Computational simulations provide a viable method for guiding mathematically-based area source management decisions and enhancing monitoring of water volume and quality. With the help of in situ monitoring and model predictions, research into the characteristics of urban area source pollution and its aquatic environmental response has expanded significantly [10]. The applicability of models such as SWAT, SWMM, MOUSE, and HSPF to area source pollution simulations can be determined by the features of the study area, simulation scale, data quality, research objective, and other factors [11,12,13,14,15]. Nonetheless, these models seldom consider the characteristics of the river course, resulting in a failure to model movements and changes therein. Other hydraulic and water quality models, including EFDC, DELFT3D, and AQUATOX, do not consider the hydrodynamic and water quality processes in river networks [16,17,18]. The Yangtze River Delta in China has the highest population density of any river network area in China. It is also highly commercially developed, with a large proportion of impervious surfaces. In such areas, it is essential to preserve river network water resources and manage environmental water quality [19,20]. Due to the difficulty of simulating all moisture, hydrodynamic, and water quality factors in a single model, it is necessary to link many models within an integrated framework.

As an area source pollution simulation model with modules for precipitation, SWMM has hydrological, hydraulic, and water quality simulation capabilities. Numerous applications exist for modeling systems based on the SWMM model. This is primarily because the model can simulate pipeline network confluence and is applied in pipeline network overflow pollution assessments, storm flood control, waterlogging warnings, and so on [21,22]. The SWMM has been thoroughly investigated and utilized in urban regional system modeling by changing river courses to pipelines. However, due to its inability to model convection-diffusion and biochemical processes, the application of SWMM in the management of river water environmental quality has been limited [23,24]. With growing demand for simultaneous modeling capability of hydrological processes and water quality features in river systems, the water volume and water quality simulation potential of the MIKE11 model have improved greatly. Based on the connection between the hydrodynamic module and multiple ecological modules, simulations of the destiny and transmission of water quality components are widely employed in investigations of the water quality of river networks [25,26,27].

Previous studies have widely documented the ability of coupled hydrodynamic-water environment models for urban area source pollution control. Wang et al. [28] studied the effects of land use and land cover changes on urban and non-urban non-point pollution with the SWAT and SWMM models. In order to analyze the effects of non-point source pollution on Baiyangdian Lake, Li et al. [6] determined the characteristics and development trends of water pollution using SWMM and a one-dimensional water quality model. However, there is still a lack of research focusing on water quality assessments and modeling in bustling cities, i.e., those characterized by rapid urbanization and artificial river channels.

In this study, we examined a typical river network region in the Yangtze River Delta, China. For the river network region, a combined model was established using a hydrological model (SWMM) and an environmental water quality model (MIKE11). A relationship between meteorological data and the concentration of pollutants was observed in one section of the river. This study provides assistance for the comprehensive management of regional urban area source pollution and the improvement of the water quality of river networks by investigating the relationship between urban area source pollution and environmental water quality.

## 2. Materials and Methods

### 2.1. Target Area

**Figure 1:**
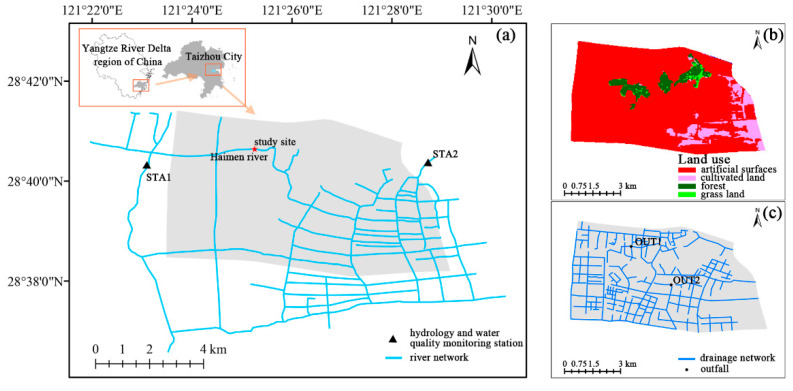
Map of the target area (**a**), land-use type (**b**), and distribution of the pipeline network (**c**).

Downtown Taizhou, Zhejiang is located in the southeast part of the Yangtze River Delta, China. Taizhou is a typical city which is threatened by urban area source pollution (Figure 1). Located in the northern portion of Wenhuang Plain, it has a water area of 59.24 km^2^, and its major landform is coastal alluvial plain. Its average annual precipitation is 1563 mm, with 75–85% being concentrated in April–October. The drainage system is obsolete, and the separation of sewer and stormwater systems is incomplete. These problems are compounded by an increase in the urban population. Pollution from urban areas is also degrading the river network’s aquatic ecosystem.

### 2.2. Model Establishment

#### 2.2.1. SWMM Model

The SWMM model was designed by the U.S. Environmental Protection Agency (EPA) as a dynamic rainfall-runoff simulation software with functions for hydrological, hydraulic, and water quality processes.

The hydrological and hydrodynamic module includes surface runoff and pipeline convergence. A SWMM model was established by dividing the target area into several sub-catchment areas, and surface flow generation and catchment process calculations were performed for each sub-catchment area. Sub-catchment areas were divided into three sections: impervious areas, impervious areas without depression storage, and impervious areas with depression storage [29]. Based on the distribution of main pipeline network inspection wells in the target area, the Tyson polygon method in the ArcGIS 10.1 neighborhood analysis tool was used to divide the catchment areas into 152 sub-catchment areas, and the administrative boundary was used to manually adjust the scope of each area. The SWMM model uses the Horton formula method, the Green-Ampt formula technique, and other flow-production simulation methods. Because the Horton model is especially suitable for urban areas, it was used for the infiltration study [30,31,32,33]. Confluence was calculated using the nonlinear reservoir approach. The SWMM model includes three calculation methods for the confluence model of a pipeline: steady flow, moving wave, and dynamic wave. The dynamic wave approach is based on the entire 1D Saint-Venant flow equation [34] and is the most applicable for such research; as such, was selected for this study.

According to land-use type, the water quality module defines the surface pollutant accumulation and erosion models and simulates the growth, flushing, transportation, and processing of pollutants in surface runoff [21,35]. This study utilized both the exponential function scour model and the saturation function accumulation model. COD_Mn_, TP, and ammonia nitrogen were selected as runoff pollution indices

#### 2.2.2. MIKE11 Model

As part of the MIKE series, the MIKE11 Model is a comprehensive 1D river course and river network simulation software developed by the Danish Water Research Institute (DHI). It is primarily used for the simulation of hydrology, hydraulics, water quality, and sediment transport in irrigation systems, estuaries, rivers, and other inland waters [36]. The hydrodynamic module of MIKE 11 assumes that water is an incompressible 1D homogeneous fluid. It employs the six-point Abt-Ionescu implicit difference scheme and solves 1D unsteady Saint Venant equations by the catch-up method. The water level, flow, velocity, and other hydraulic data of the river course were calculated to provide a basis (e.g., continuity and momentum equations) for the simulation of pollutant diffusion transfer [37]. The MIKE 11 convection-diffusion module utilizes the law of mass conservation for the convection-diffusion equation to simulate the migration of pollutants in water [38].

### 2.3. Data Source

Land use data with 30-m resolution were acquired from GLOBELAND30 Global Geographic Information Public Goods (http://www.globallandcover.com/, accessed on 16 May 2022). In the target area, land use types are primarily split into three categories: synthetic surface, grassland, and forest; the former is the predominant land use in the area of interest (67%). The national meteorological science data center (http://data.cma.cn/, accessed on 10 December 2018) provides hourly meteorological data including precipitation, temperature, and wind speed. Two hydrology and water quality monitoring stations were set up in the target area (STA1 and STA2). Water level and quality monitoring data were used to calibrate the water quality simulation model of the river network. Pollution data from sewage treatment plants and other point-sources and attribute information from the pipeline network were acquired from reports by the local water conservation, environmental protection, and housing development departments. Other hydraulics for the OUT1 and OUT2 pipeline networks were used to calibrate SWMM runoff debris and water quality in the target area.

### 2.4. Framework for Model Coupling and Analysis

**Figure 2:**
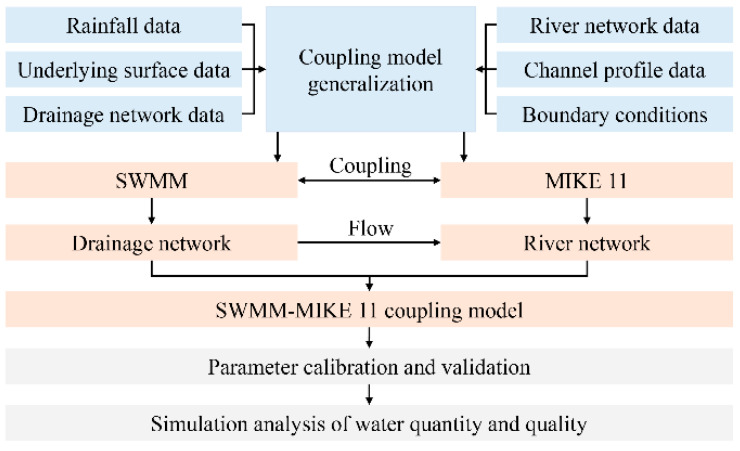
Coupling scheme of the SWMM and MIKE11 models.

The SWMM and MIKE11 models were coupled using an output–input approach (Figure 2). SWMM was initially used to generalize each catchment partition and pipeline network. Then, the river course was summarized using the MIKE11 model. The pipeline network and river models were coupled with the pipeline network discharge ports as nodes. Rainfall from the watershed is first gathered by a pipeline network and then goes into the river through each discharge point. A pipeline network model was used to simulate variations in rainfall and pollutant concentration. The application converts the water quality from discharge ports and uses the resulting data to generate a river network model. Using this framework, different hydrological conditions were simulated to analyze the relationship between urban area source pollution and water quality in a river network. To calibrate and verify the coupled model, SWMM can be calibrated and verified first; then, the runoff results calculated by SWMM can be imported into MIKE 11 as boundary conditions, and finally, the MIKE 11 model can be calibrated and verified.

The Morris screening method [39] was adopted to identify the parameters that have significant impacts on the model output, so as to improve the efficiency of the model calibration and verification. Mannings N for impervious areas, mannings N for pervious areas, depth of depression storage on impervious areas, depth of depression storage on pervious areas, percent of impervious area with no depression storage, maximum rate on the Horton infiltration curve, minimum rate on the Horton infiltration curve, decay constant for the Horton infiltration curve, and time for a fully saturated soil to completely day were the sensitivity parameters of the runoff process in the SWMM model. Maximum possible buildup per unit of normalizer variable, time exponent for power buildup, washoff coefficient, and runoff exponent in washoff function were the sensitivity parameters of the runoff pollution process in the SWMM model. The river course roughness and comprehensive attenuation coefficients were the sensitivity parameters in the MIKE11 model.

The sequential uncertainty fitting method [40] was used to determine the optimal ranges and values of the parameters through iterative calculations, and the optimal values of these parameters were introduced into the model through internal adjustments. The Nash-Sutcliffe efficiency coefficient (NSE) and relative error (RE) were employed to assess the quality and precision of the simulation results [41]. NSE can reflect hydrological model simulation results: values greater than 0.50 indicate that the simulation quality is sufficient, while values near 1 suggest that the simulation quality is high. RE values less than 0.25 indicate that the simulation error is within an acceptable range.

## 3. Results

### 3.1. Simulation of Urban Area Source Pollution Loads

**Figure 3:**
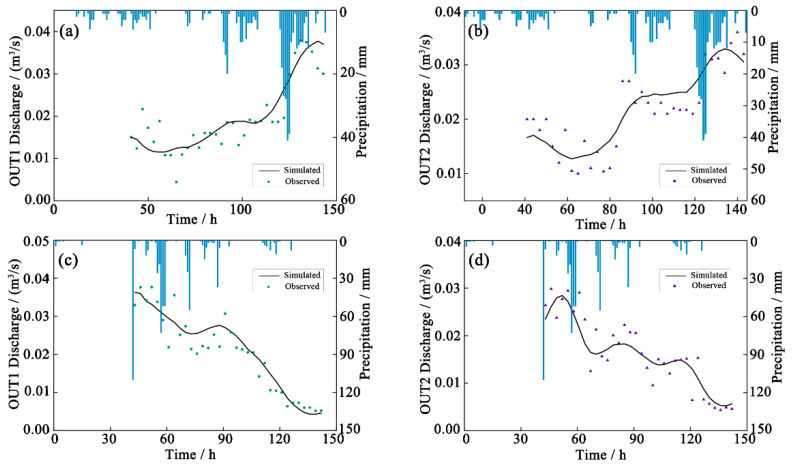
Calibration (**a**,**b**) and validation (**c**,**d**) results for runoff in the SWMM model.

**Figure 4:**
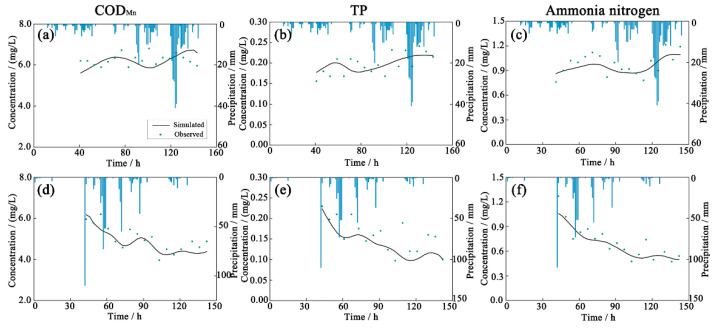
Calibration (**a**–**c**) and validation (**d**–**f**) results for water quality at the OUT1 drainage outlet in the SWMM model.

The SWMM model parameters were calibrated for the target area using the rainfall scenario from 11 A.M. on 3 January to 11 P.M. on 8 January 2018. The model parameters were then validated using the rainfall scenario from 0 A.M. on 19 June to 5 A.M. on 24 June 2018. The results of the model calibration and verification (Figure 3 and Figure 4) demonstrated that model outputs corresponded well with the measured flow and pollutant concentrations. NSE exceeded 0.8, and absolute RE was below 20%. NSE values of COD_Mn_, TP, and ammonia nitrogen varied between 5% and 16%, indicating that the SWMM model had accurately simulated drainage system runoff and water quality in the target area.

Table 1 shows the hydrological and hydraulic parameters of the SWMM model for the area of interest. Table 2 shows the buildup and washoff parameter values for various land-use patterns. For synthetic surfaces, the results were insignificant for COD_Mn_, TP, and ammonia nitrogen, with maximum saturations of 20, 0.4, and 2 kg/ha, respectively. For scour coefficients of 0.002, 0.004, and 0.004, the scour indices were 1.2, 1.3, and 1.3, respectively.

Pollutant concentrations and flow at the OUT1 drainage outlet were then simulated under the rainfall scenario from 0 A.M. on 19 June to 5 A.M. on 24 June 2018. COD_Mn_, TP, and ammonia nitrogen were 39.3, 1.2, and 5.7 kg, respectively, and RE values were 1%, 9%, and 3%, respectively, indicating that the SWMM model simulation was of sufficient quality. According to a comprehensive simulation of annual meteorological precipitation data from 2018, the annual inflow loads of COD_Mn_, TP, and ammonia nitrogen from urban area sources due to rainfall were 29.8, 0.9, and 4.8 t, respectively.

### 3.2. Determination of Model Parameters for an Environmental Water Quality Assessment of a River Network

**Figure 5:**
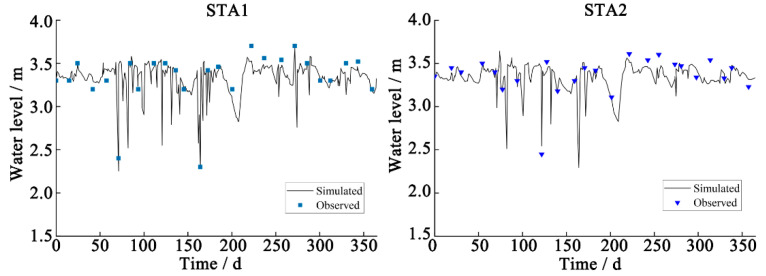
Water level calibration results of the MIKE11 model for the target area.

**Figure 6:**
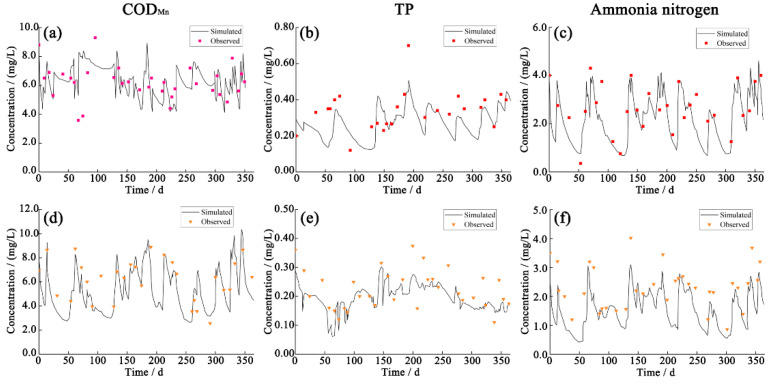
Water quality calibration results at STA1 (**a**–**c**) and STA2 (**d**–**f**) of the MIKE11 model.

Hydrodynamics and water volume are necessary inputs for the simulation of water quality using the river network model. In this study, 20 river courses were generalized. The pollution load was based on data from 2018. Urban area source pollution load was calculated using the SWMM model. The river course roughness and comprehensive attenuation coefficient of pollutants were calibrated using measurements of water level and quality, collected by monitoring stations in the area of interest. The RE values for simulated water levels remained below 10% (Figure 5), while RE values for simulated COD_Mn_, TP, and ammonia nitrogen concentrations at eight sites remained below 25% (Figure 6), suggesting that model simulations were accurate. According to model simulation, the river course roughness was 0.025~0.035, while the comprehensive attenuation coefficients of COD_Mn_, TP, and ammonia nitrogen were 0.08–0.12, 0.05–0.25, and 0.08–0.10 d^−1^, respectively.

### 3.3. Relationship between Urban Area Source Pollution and Water Environment Quality

Haimen River, a typical plain channel in China’s Yangtze River Delta that runs through the central urban area, was used for the next set of simulations. Three distinct rainfall events were selected: light rain, moderate rain, and heavy rain. Water quality changes at the OUT1 drainage outlet of a pipeline network into an estuary were simulated under various rainfall scenarios.

**Figure 7:**
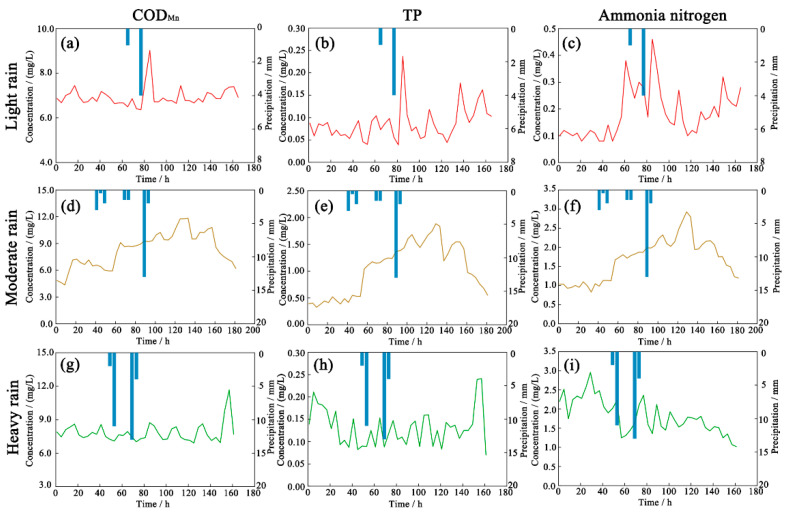
Pollution variation trends under light (**a**–**c**), moderate (**d**–**f**), and heavy rain (**g**–**i**).

Under the rainfall scenario from 2 P.M. on 25 March to 4 P.M. on 26 March 2018, total rainfall duration was 12 h and total precipitation was 5 mm, which corresponds to light rain (Figure 7a–c). There was a time lag between the start of the rainfall event and the change in pollutant concentrations. COD_Mn_, TP, and ammonia nitrogen continued to climb significantly after precipitation stopped, with values peaking after 8 h at a maximum COD_Mn_ concentration of 9.02 mg/L. The concentration of TP was 0.24 mg/L, while that of ammonia nitrogen was 0.46 mg/L. Sixteen hours after precipitation, TP and ammonia-nitrogen concentrations steadily fell to pre-rainfall levels. In the subsequent 4 h, the concentration of COD_Mn_ returned to pre-rainfall levels. The total duration of the effect of rainfall was 24 h.

Under the rainfall scenario between 4 P.M. on 16 July to 8 P.M. on 18 July 2018, the total rainfall duration was 52 h and the total precipitation was 23.5 mm, which corresponds to moderate rain. As depicted in Figure 7d–f, there was a time lag between the start of the rainfall event and the change in pollutant concentrations, similar to that observed in the light rain scenario. As rainfall continued, pollutant concentrations increased and peaked 40 h after the onset of rainfall, with a COD_Mn_ concentration of 11.76 mg/L, a TP concentration of 1.89 mg/L, and an ammonia nitrogen concentration of 8.78 mg/L. Values returned to their pre-rainfall levels after 52 h. The rainfall event no longer influenced water quality 92 h after precipitation stopped. During this period, the diluting effect of moderate rain did not exert a significant influence.

Under the rainfall scenario between 0 A.M. on 15 September to 0 A.M. on 16 September 2018, the total duration of rainfall was 24 h and the total precipitation was 30 mm, which corresponds to heavy rain. As depicted in Figure 7g–i, the pollutant concentration change was relatively steady in the simulations. As rainfall progressed, the concentration of ammonia-nitrogen fell.

In summary, under the scenario of low rainfall, the effects of rain on environmental water quality in river courses were delayed and transient. Under moderate rainfall, runoff pollution compounded the overflow pollution of the pipe network. Environmental water quality was more affected under light and heavy rain, and the effects lasted longer than under moderate rain. Under the scenario of heavy rain, the diluting effect of rain dominated.

## 4. Discussion

Downtown Taizhou, Zhejiang Province, is a typical river network area with a high proportion of impervious surfaces. Inferior hydrodynamic circumstances caused by the superposition of a plain landscape on a river course make accurate simulations of the water pollutant diffusion process more challenging [42]; in this respect, the SWMM hydrological model alone may not be able to meet all of the requirements. Simulations of water volume and quality combining SWMM and MIKE11 yielded better results. For the SWMM model, the NSEs of simulated flow at pipeline network drains exceeded 0.8 and absolute REs of simulated flow and pollutant concentrations were under 20%. The simulation quality requirements for drainage system runoff and water quality were satisfied for the target region. Likewise, the simulated REs of COD_Mn_, TP, and ammonia nitrogen concentrations were within 25%, indicating that the water quality predictions were highly accurate. This study employed the output–input coupling system, which is a practical method for the exhaustive simulation of water volume and quality.

The integrated simulation of water quantity and quality requires a comprehensive description of hydrological, chemical, and biogeochemical processes. Existing models, such as simple export coefficient models [43] and complex process-based models [44], have been derived for different research and have shown various capabilities and degrees of complexity. Therefore, there is always a tradeoff between model structure, computation cost, and modeling accuracy. The advantages of the “output–input” scheme in a coupled modeling system have become evident: such a model greatly assists in overcoming multidisciplinary problems on various scales and provides improved performance. The difficulty of coupling open source and commercial software is resolved using an output–input method. The output–input coupling scheme adopted in this study did not consider the impact of other hydrological factors on river pollutants, including the impacts of pipeline sediment migration and transformation; this may have affected the simulation accuracy. When the coupling model is applied to an actual project, the land type, the complexity of the river and underground pipe network system, and the incompleteness of the monitoring conditions must be considered, as these variables have the potential to lead to increases in the coupling model error. In the process of building a coupling model, the timeliness and consistency of the parameter source data for the SWMM and Mike 11 models must first be ensured, and the sensitive parameters must be extracted by using satellite image map, ArcGIS, and other software and mathematical statistical analysis methods.

Water pollution is influenced by many factors, including land use, meteorological conditions, hydrological conditions, and geological conditions [45,46,47,48]. Water quality varies seasonally and is affected by different factors, albeit mainly by rainfall. Rainfall affects the hydrological cycle, the physical and chemical properties of pollutants [49], pollutant migration, and the ability of water bodies to dilute pollutants. To understand the underlying mechanism driving the variations in pollutant concentrations, this study simulated environmental water quality measurements in a river course under different rainfall scenarios. Our results provide useful guidance for urban planning and the management of urban waterways in the future.

Simulations of pollutant concentrations in a river course under scenarios of light, moderate, and heavy rain revealed that for the former, there is a lag between rain entering the river course and changes in pollutant concentrations. The process of urban rainwater confluence is surface–pipe network–discharge–river channel [50]. In this study, the target region was comprised of medium-sized, economically developed coastal cities in southeast China with reasonably good pipeline network infrastructures [51,52]. Before entering the pipeline network, runoff from rainfall is blocked by green spaces and other low-impact development facilities, thus delaying its entry. Rainwater storage and interception facilities in pipeline networks can also intercept runoff, reducing the impact of runoff on river water quality. In the event of moderate rain, surface contaminants are transported rapidly into the pipeline network. The drainage system in the target area is relatively old, and rainwater and sewage are not completely separated; as such, sewage can easily merge with rainwater. Because surface runoff and sewage can enter the river immediately, river water quality is adversely affected. After precipitation, surface runoff enters the pipeline network, and pipeline flow steadily increases. When flow exceeds the transmission capacity of a pipeline network or sewage treatment facility, the pipeline network overflows and surface runoff is combined with water in the network [53,54]. The pollutant index of sewage can then significantly exceed the critical level. In some cases, pollutants contain volatile organic compounds, heavy metals, and pesticides, placing a heavy burden on the aquatic environment. River water quality can degrade rapidly, affecting ecosystem health [55]. Heavy precipitation causes drainage systems and river networks to receive a lot of runoff in a short period of time. As a result, the concentrations of water pollutants remain largely steady during such rainfall events. Overall, the concentrations of runoff pollutants and the runoff volume entering the water body are the factors that most directly influence the water quality.

China’s investments in maintaining a high quality of urban water have paid off in the past decade. Despite this, area source pollution continues to be the predominant cause of the deterioration in the environmental quality of urban waterways. Due to the small amount of runoff and low water capacity of the river network, controlling urban area source pollution can stabilize and improve river water quality [9]. Therefore, the following suggestions are provided:

(1) Source control is the cornerstone of urban area source management; this includes removing sediment from drainage pipeline networks, investigating and improving sewage outlets, etc. The optimization of low-impact urban development measures can control the heavy pollution present in initial stormwater runoff. This would be an effective way to alleviate urban non-point source pollution. Green infrastructure, biological retention systems, and pervious pavements mainly rely on the natural purification effect of vegetation, soil, and wetlands. Cities can absorb and release rain using a combination of natural and manmade methods [56,57].
(2) The current coverage of urban environmental quality monitoring stations is inadequate and cannot accurately convey urban environmental quality [58,59]. Based on the present surface water and urban environmental quality monitoring network, urban area source pollution monitoring infrastructure should be established in high-load areas. Monitoring should be conducted during key periods, such as the flood season, and in key areas near surface water. Using ground observations and quantitative analyses, the load and spatio-temporal evolution of urban area source pollution should be estimated.
(3) Application-integrated smart manhole covers, water quality sensors, and 5G Internet of Things will be relevant tools for pollution monitoring and reduction. Additionally, long-term, continuous, autonomous monitoring and data exchange of drainage pipeline network pollution should be conducted [60,61,62,63]. A coupling model of urban hydrology, water quality, and hydrodynamic, non-point source pollution which is suitable for different scales should be developed, improved, and popularized to better analyze the characteristics of urban non-point source pollution and trace the sources pollutants.

## 5. Conclusions

This study examined a city in the river network of the Yangtze River Delta, China. Coupled hydrodynamic and hydrodynamic water quality models (SWMM and MIKE11) were used to investigate the relationship between urban area source pollution and environmental water quality. The difficulty of coupling open source and commercial software was resolved using an “output–input” method. Under conditions of light rain, the effect of precipitation on the water quality in the river network was found to be delayed and transitory. Under the scenario of moderate rain, precipitation had a significant impact on the water quality of the river course. With moderate rain, the water quality in the river was worse than that of light rain and heavy rain. Under conditions of heavy rain, excess water serves as a diluting agent. Pollution overflow from pipeline networks resulting from rainfall is a severe issue that requires attention. Source control, process interception, terminal treatment, and ecological remediation can all contribute to urban area source pollution control with the goal of enhancing water quality.

## Figures and Tables

**Table 1:** Hydrological and hydraulic parameters in the SWMM model of the target area.

Parameter Symbol	Parameter Meaning	Value Range
*N-Imperv*	Mannings N for impervious areas	0.005~0.020
*N-Perv*	Mannings N for pervious areas	0.50~0.80
*Dstore-Imperv*	Depth of depression storage on impervious areas/mm	0.5~5.0
*Dstore-Perv*	Depth of depression storage on pervious areas/mm	2~10
*%Zero-Imperv*	Percent of impervious areas with no depression storage/%	25~70
*Max. Infil. Rate*	Maximum rate on the Horton infiltration curve/(mm/h)	40~80
*Min. Infil. Rate*	Minimum rate on the Horton infiltration curve/(mm/h)	1~5
*Decay Constant*	Decay constant for the Horton infiltration curve/(1/h)	2~6
*Dry Time*	Time for a fully saturated soil to completely day/h	2~8

**Table 2:** Water quality parameters in the SWMM model of the target area.

Types of Land Use	Pollutant Index	Buildup Parameters		Washoff Parameters	
		*Max. Buildup*/(kg/ha)	*Power/Sat. Constant*/(d)	*Coefficient*	*Exponent*
Artificial Surfaces	COD_Mn_	20	1.5	0.002	1.2
	TP	0.4	1.6	0.004	1.3
	ammonia nitrogen	2	1.9	0.004	1.3
Cultivated Land	COD_Mn_	40	10	0.004	1.6
	TP	0.6	10	0.002	1.7
	ammonia nitrogen	40	10	0.001	1.6
Forest and Grass Land	COD_Mn_	20	10	0.004	1.5
	TP	10	1	0.001	1.5
	ammonia nitrogen	10	1	0.002	1.6

The abbreviations are as follows: *Max. Buildup*—maximum possible buildup per unit of normalizer variable/(kg/ha); *Power/Sat. Constant*—time exponent for power buildup or half-saturation constant for saturation buildup/(d); *Coefficient*—washoff coefficient or Event Mean Concentration (EMC); *Exponent*—runoff exponent in the washoff function.

## Data Availability

The data and software generated or used during the study appear in the submitted article.

## References

[1] Zhou C., Li H. (2022). Analysis on the objectives and measures of comprehensive treatment of human settlement water environment under the background of rural revitalization. Fresen. Environ. Bull..

[2] Yang J., Liang J., Yang G., Feng Y., Ren G., Ren C., Han X., Wang X. (2020). Characteristics of Non-Point Source Pollution under Different Land Use Types. Sustainability.

[3] Zhang N., Zhang Q., Li Y., Zeng M., Li W., Chang C., Xu Y., Huang C. (2020). Effect of Groundcovers on Reducing Soil Erosion and Non-Point Source Pollution in Citrus Orchards on Red Soil Under Frequent Heavy Rainfall. Sustainability.

[4] Chen L., Xu J., Wang G., Liu H., Zhai L., Li S., Sun C., Shen Z. (2018). Influence of rainfall data scarcity on non-point source pollution prediction: Implications for physically based models. J. Hydrol..

[5] Ma Y., Hao S., Zhao H., Fang J., Zhao J., Li X. (2018). Pollutant transport analysis and source apportionment of the entire non-point source pollution process in separate sewer systems. Chemosphere.

[6] Li C., Zheng X., Zhao F., Wang X., Cai Y., Zhang N. (2017). Effects of Urban Non-Point Source Pollution from Baoding City on Baiyangdian Lake, China. Water.

[7] Huang L., Han X., Wang X., Zhang Y., Yang J., Feng A., Li J., Zhu N. (2022). Coupling with high-resolution remote sensing data to evaluate urban non-point source pollution in Tongzhou, China. Sci. Total Environ..

[8] Fu C., Su J., Zhao H., Li Q. (2020). Estimation of urban non-point source pollution load in upper reaches of Zhanghe River based on GIS. Water Resour. Prot..

[9] Zong M., Hu Y., Liu M., Li C., Wang C., Liu J. (2021). Quantifying the Contribution of Agricultural and Urban Non-Point Source Pollutant Loads in Watershed with Urban Agglomeration. Water.

[10] Ji H., Peng D., Fan C., Zhao K., Gu Y., Liang Y. (2022). Assessing effects of non-point source pollution emission control schemes on Beijing’s sub-center with a water environment model. Urban Clim..

[11] Zhou C.W., Yu L.F., Zhou Y., Yan L.B. (2019). Hydrological and ecological effect of caohai watershed regulation project based on swat model. Appl. Ecol. Environ. Res..

[12] Zhang X., Chen P., Dai S., Han Y. (2022). Analysis of non-point source nitrogen pollution in watersheds based on SWAT model. Ecol. Indic..

[13] Kim T.G., Choi K. (2020). A study on water quality change by land use change using HSPF. Environ. Eng. Res..

[14] Adu J.T., Kumarasamy M.V. (2018). Assessing Non-Point Source Pollution Models: A Review. Pol. J. Environ. Stud..

[15] Li Q., Wang F., Yu Y., Huang Z., Li M., Guan Y. (2019). Comprehensive performance evaluation of LID practices for the sponge city construction: A case study in Guangxi, China. J. Environ. Manag..

[16] Zhao F., Li C., Chen L., Zhang Y. (2018). An Integrated Method for Accounting for Water Environmental Capacity of the River-Reservoir Combination System. Water.

[17] Da Silva Burigato Costa C.M., Marques L.D.S., Almeida A.K., Leite I.R., de Almeida I.K. (2019). Applicability of water quality models around the world-a review. Environ. Sci. Pollut. Res..

[18] Da Silva Burigato Costa C.M., Leite I.R., Almeida A.K., de Almeida I.K. (2021). Choosing an appropriate water quality model—A review. Environ. Monit. Assess..

[19] Xu G., Li A., Yang Q., Chi J. (2022). Spatiotemporal change in the river network in rapidly urbanized plain regions of the Yangtze River Delta in China. River Res. Appl..

[20] Liao Y., Li Y., Shu J., Wan Z., Jia B., Fan Z. (2021). Water Transparency Prediction of Plain Urban River Network: A Case Study of Yangtze River Delta in China. Sustainability.

[21] Gironas J., Roesner L.A., Rossman L.A., Davis J. (2010). A new applications manual for the Storm Water Management Model (SWMM). Environ. Modell. Softw..

[22] Hsu M.H., Chen S.H., Chang T.J. (2000). Inundation simulation for urban drainage basin with storm sewer system. J. Hydrol..

[23] Latifi M., Rakhshandehroo G., Nikoo M.R., Sadegh M. (2019). A game theoretical low impact development optimization model for urban storm water management. J. Clean. Prod..

[24] Yang Y., Sun L., Li R., Yin J., Yu D. (2020). Linking a Storm Water Management Model to a Novel Two-Dimensional Model for Urban Pluvial Flood Modeling. Int. J. Disaster Risk Sci..

[25] Tang C., Yi Y., Yang Z., Cheng X. (2014). Water pollution risk simulation and prediction in the main canal of the South-to-North Water Transfer Project. J. Hydrol..

[26] Ahmed F. (2010). Numerical modeling of the Rideau Valley Watershed. Nat. Hazards.

[27] Prucha B., Graham D., Watson M., Avenant M., Esterhuyse S., Joubert A., Kemp M., King J., le Roux P., Redelinghuys N. (2016). MIKE-SHE integrated groundwater and surface water model used to simulate scenario hydrology for input to DRIFT-ARID: The Mokolo River case study. Water SA.

[28] Wang G., Yang K., Yang Y., Zhang S. (2017). Coupling natural and human processes to simulate changes in the water environment in the Dianchi Lake basin, China. Geosyst. Eng..

[29] Bisht D.S., Chatterjee C., Kalakoti S., Upadhyay P., Sahoo M., Panda A. (2016). Modeling urban floods and drainage using SWMM and MIKE URBAN: A case study. Nat. Hazards.

[30] Rosa D.J., Clausen J.C., Dietz M.E. (2015). Calibration and Verification of SWMM for Low Impact Development. J. Am. Water Resour. Assoc..

[31] Ren X., Hong N., Li L., Kang J., Li J. (2020). Effect of infiltration rate changes in urban soils on stormwater runoff process. Geoderma.

[32] Moore M.F., Vasconcelos J.G., Zech W.C. (2017). Modeling Highway Stormwater Runoff and Groundwater Table Variations with SWMM and GSSHA. J. Hydrol. Eng..

[33] Tsai L., Chen C., Fan C., Lin J. (2017). Using the HSPF and SWMM Models in a High Pervious Watershed and Estimating Their Parameter Sensitivity. Water.

[34] Chen X., Ji P., Wu Y., Zhao Y., Zeng L. (2017). Coupling simulation of overland flooding and underground network drainage in a coastal nuclear power plant. Nucl. Eng. Des..

[35] Lee S., Yoon C., Jung K.W., Hwang H.S. (2010). Comparative evaluation of runoff and water quality using HSPF and SWMM. Water Sci. Technol..

[36] Villuri V.G.K., Pasupuleti S., Jain K., Gairola A., Singh R.K. (2018). Hydrodynamic simulation of a cloudburst event in Asi Ganga Valley of Indian Himalayan region using MIKE11 and GIS techniques. Mausam.

[37] Bu J., Li C., Wang X., Zhang Y., Yang Z. (2020). Assessment and prediction of the water ecological carrying capacity in Changzhou city, China. J. Clean. Prod..

[38] Kubrak J., Kiczko A., Kubrak E. (2021). Case Study: Forecasting the Lower Vistula Bed Deformation without and with Development of Dam Cascade. Water.

[39] Huang J., Wen J., Wang B., Hinokidani O. (2020). Parameter sensitivity analysis for a physically based distributed hydrological model based on Morris’ screening method. J. Flood Risk Manag..

[40] Singh A., Jha S.K. (2021). Identification of sensitive parameters in daily and monthly hydrological simulations in small to large catchments in Central India. J. Hydrol..

[41] Moriasi D.N., Arnold J.G., Van Liew M.W., Bingner R.L., Harmel R.D., Veith T.L. (2007). Model evaluation guidelines for systematic quantification of accuracy in watershed simulations. Trans. ASABE.

[42] Qi Z., Ye X., Zhang H., Yu Z. (2014). Land fragmentation and variation of ecosystem services in the context of rapid urbanization: The case of Taizhou city, China. Stoch. Environ. Res. Risk Assess..

[43] Wang W., Chen L., Shen Z. (2020). Dynamic export coefficient model for evaluating the effects of environmental changes on non-point source pollution. Sci. Total Environ..

[44] Garcia-Alba J., Barcena J.F., Ugarteburu C., Garcia A. (2019). Artificial neural networks as emulators of process-based models to analyse bathing water quality in estuaries. Water Res..

[45] Guo D., Lintern A., Webb J.A., Ryu D., Liu S., Bende-Michl U., Leahy P., Wilson P., Western A.W. (2019). Key Factors Affecting Temporal Variability in Stream Water Quality. Water Resour. Res..

[46] Lintern A., Webb J.A., Ryu D., Liu S., Bende-Michl U., Waters D., Leahy P., Wilson P., Western A.W. (2018). Key factors influencing differences in stream water quality across space. WIREs Water.

[47] Xu G., Li P., Lu K., Zhan T., Zhang J., Ren Z., Wang X., Yu K., Shi P., Cheng Y. (2019). Seasonal changes in water quality and its main influencing factors in the Dan River basin. Catena.

[48] Bojarczuk A., Jelonkiewicz L., Lenart-Boron A. (2018). The effect of anthropogenic and natural factors on the prevalence of physicochemical parameters of water and bacterial water quality indicators along the river Biaka, southern Poland. Environ. Sci. Pollut. Res. Int..

[49] Casas G., Martinez-Varela A., Vila-Costa M., Jimenez B., Dachs J. (2021). Rain Amplification of Persistent Organic Pollutants. Environ. Sci. Technol..

[50] Meilvang M.L. (2021). From rain as risk to rain as resource: Professional and organizational changes in urban rainwater management. Curr. Sociol..

[51] Tian J., He G. (2020). Optimization design method for urban sewage collection pipe networks. Water Sci. Technol..

[52] Tian J., Cheng J., Gong Y. (2018). Optimization of municipal pressure pumping station layout and sewage pipe network design. Eng. Optimiz..

[53] Peche A., Graf T., Fuchs L., Neuweiler I. (2019). Physically based modeling of stormwater pipe leakage in an urban catchment. J. Hydrol..

[54] El-Housni H., Duchesne S., Mailhot A. (2019). Predicting Individual Hydraulic Performance of Sewer Pipes in Context of Climate Change. J. Water Resour. Plan. Manag..

[55] Yin Z., Duan R., Li P., Li W. (2021). Water quality characteristics and health risk assessment of main water supply reservoirs in Taizhou City, East China. Hum. Ecol. Risk Assess..

[56] Sharma R., Malaviya P. (2021). Management of stormwater pollution using green infrastructure: The role of rain gardens. WIREs Water.

[57] Liu A., Jiang Y., Dockko S., Guan Y. (2015). Characterizing stormwater treatment efficiency at the laboratory scale for effective rain garden design. Desalin. Water Treat..

[58] Chen P. (2019). Visualization of real-time monitoring datagraphic of urban environmental quality. EURASIP J. Image Video Process..

[59] Wei W., Zhang Y., Wan G. (2020). Research on Surface Water Quality in China-based on Observation of 148 Automatic Monitoring Stations. J. Coast. Res..

[60] Kozak C., Leithold J., Do Prado L.L., Knapik H.G., de Rodrigues Azevedo J.C., Braga S.M., Fernandes C.V.S. (2021). Adaptive monitoring approach to assess dissolved organic matter dynamics during rainfall events. Environ. Monit. Assess..

[61] Zhuang Y., Wen W., Ruan S., Zhuang F., Xia B., Li S., Liu H., Du Y., Zhang L. (2022). Real-time measurement of total nitrogen for agricultural runoff based on multiparameter sensors and intelligent algorithms. Water Res..

[62] Chen B., Mu X., Chen P., Wang B., Choi J., Park H., Xu S., Wu Y., Yang H. (2021). Machine learning-based inversion of water quality parameters in typical reach of the urban river by UAV multispectral data. Ecol. Indic..

[63] Strokal M., Spanier J.E., Kroeze C., Koelmans A.A., Floerke M., Franssen W., Hofstra N., Langan S., Tang T., van Vliet M.T.H. (2019). Global multi-pollutant modelling of water quality: Scientific challenges and future directions. Curr. Opin. Environ. Sustain..

